# Anti-Neu5Gc and anti-non-Neu5Gc antibodies in healthy humans

**DOI:** 10.1371/journal.pone.0180768

**Published:** 2017-07-17

**Authors:** Bingsi Gao, Cassandra Long, Whayoung Lee, Zhongqiang Zhang, Xiaotian Gao, Doug Landsittel, Mohamed Ezzelarab, David Ayares, Yuliang Huang, David K. C. Cooper, Yi Wang, Hidetaka Hara

**Affiliations:** 1 Thomas E. Starzl Transplantation Institute, University of Pittsburgh, Pittsburgh, PA, United States of America; 2 Center for Kidney Transplantation, Second Affiliated Hospital of the University of South China, Hengyang, Hunan, China; 3 Department of Obstetrics and Gynecology, Second Affiliated Hospital of the University of South China, Hengyang, Hunan, China; 4 Department of General Surgery, Second Xiangya Hospital of the Central South University, Changsha, Hunan, China; 5 Department of Biostatistics, University of Pittsburgh, Pittsburgh, PA, United States of America; 6 Department of Biostatistics and Clinical and Translational Science, University of Pittsburgh, Pittsburgh, PA, United States of America; 7 Revivicor, Blacksburg, VA, United States of America; Universidade de Sao Paulo, BRAZIL

## Abstract

Our group previously investigated the levels of anti-Gal and anti-nonGal IgM and IgG in a cohort of 75 healthy humans of various backgrounds, and found some significant differences related to factors such as age, gender, ABO blood group, diet, vaccination history, and geographic location during childhood. We have now expanded our cohort (n = 84) to investigate the levels of anti-Neu5Gc and anti-nonGal/nonNeu5Gc antibodies in healthy humans. Anti-nonGal and anti-nonGal/nonNeu5Gc human IgM and IgG binding to pRBCs and pAECs from GTKO/CD46 and GTKO/CD46/Neu5GcKO pigs were measured by flow cytometry. Anti-Gal and anti-Neu5Gc IgM and IgG levels were measured by ELISA. In summary, (i) the great majority (almost 100%) of humans had anti-Neu5Gc IgM and IgG antibodies that bound to pAECs and approximately 50% had anti-Neu5Gc antibodies that bound to pRBCs, (ii) there was significantly less human antibody binding to pig cells that did not express either Gal or Neu5Gc compared with those that did not express Gal alone, (iii) the levels of both IgM and IgG binding to GTKO/CD46/Neu5GcKO pRBCs and pAECs were low, (iv) the level of anti-Neu5Gc IgG was higher in men than women, (v) the level did not change with age or diet, and there was some variability associated with (vi) previous vaccination history and (vii) the geographic region in which the individual spent his or her childhood. Our study confirms that human antibody binding to RBCs and AECs from GTKO/CD46/Neu5GcKO pigs is greatly reduced compared to binding to GTKO/CD46 cells. However, all humans appear to have a low level of antibody that binds to pAECs that is not directed to either Gal or Neu5Gc. Our findings require consideration in planning clinical trials of xenotransplantation.

## Introduction

Because of inactivation of the α1,3-galactosyltransferase gene, humans do not express galactose-α1,3-galactose (Gal) [1]. As a result, humans develop natural antibodies directed to this oligosaccharide that is expressed on many wild-type (i.e., genetically-unmodified) pig cells [1–4]. Binding of primate anti-Gal antibodies to pig Gal epitopes results in hyperacute rejection (defined as graft failure within 24 hours after transplantation) or early graft failure [5–7]. The availability of pigs homozygous for α1,3-galactosyltransferase gene-knockout (GTKO) [8] has enabled pig-to-baboon organ transplantation to be carried out in the absence of Gal epitopes [9, 10]. Recently, several groups have reported significantly longer xenograft survival using genetically-engineered pigs in nonhuman primates (e.g., almost 10 months for life-supporting kidneys [11–13], and >2 years for non-life-supporting [heterotopic] hearts [14] xenografts).

Our group previously investigated the levels of anti-Gal and anti-nonGal IgM and IgG in a cohort of 75 human subjects of various backgrounds [15]. In particular, we investigated the influence of age, gender, ABO blood group, diet, history of vaccination, and geographic location during childhood on antibody levels, the major conclusions from which are summarized in **S1 Table**.

N-glycolylneuraminic acid (Neu5Gc) is an important nonGal antigen that is expressed in pigs, apes, and Old World nonhuman primates, but not in humans [16–18]. Therefore, humans develop anti-Neu5Gc antibodies (particularly of the IgG phenotype) [19]. Pigs homozygous for cytidine monophospho-N-acetylneuraminic acid hydroxylase gene-knockout (Neu5GcKO) that do not express Neu5Gc are now available [20–23]. Our group has recently shown that human antibody binding to GTKO/CD46/Neu5GcKO pig cells is significantly reduced in comparison to binding to GTKO/CD46 pig cells [22, 23], but this conclusion was drawn from a cohort of only six subjects [23].

We have now used the same 75 human sera from our previous study, with an additional 9 sera (total 84), to measure anti-nonGal, anti-Neu5Gc, and anti-nonGal/nonNeu5Gc IgM and IgG antibodies to determine variations associated with age, gender, ABO blood group, diet, history of vaccination, and geographic background.

We measured human serum IgM and IgG binding to both pig red blood cells (pRBCs; that express pig glycans but not MHC class I or II antigens [i.e., SLA]) and pig aortic endothelial cells (pAEC; that express both pig glycans and MHC class I and II antigens). We also explored whether a single measurement, e.g., of anti-nonGal antibody, could predict the presence and level of other antibodies, e.g., anti-Neu5Gc or anti-nonGal/nonNeu5Gc.

## Materials and methods

All animal care was in accordance with the Guide for the Care and Use of Laboratory Animals prepared by the National Research Council (8^th^ edition, revised 2011), and was conducted in an AAALAC-accredited facility. Protocols were approved by the University of Pittsburgh Institutional Animal Care and Use Committee (IACUC#13082323).

All *in vitro* studies using human serum or blood were approved by the Research Ethics Committee of the University of Pittsburgh. The samples were obtained in accordance with the Declaration of Helsinki. Written informed consent was obtained from each participant as per the guidelines of the Institutional Review Board of the University of Pittsburgh (IRB#0608179).

### Sources of human sera

Serum was collected from 84 healthy human volunteers of all ABO blood groups (A, B, O, AB) who had spent at least the initial 18 years of their lives in a specific region of the world **(Table 1).** (These sera included the 75 tested in our previous report [15]) Samples were collected from 56 males and 28 females, with a combined mean age of 35.6 years. None of the volunteers had a history suggesting previous exposure to pig antigens (except in the diet). Demographic details, including age, gender, ABO blood group, and dietary and vaccination histories were recorded (**Table 1**). In addition, sera were grouped according to the geographic origin of the human volunteers (i.e., Europe, Middle-East, Africa, South-West Asia, East-Asia, South America, and North America) **(Table 1)**.

**Table 1:** Age, gender, ABO group, dietary and vaccination histories, and geographic area of childhood in 84 healthy human subjects from whom sera were studied in the present report.

*N = 84; average age = 35*.*6y*		
***Age***	*20–29 = 26*.*2% (22/84); 30–39 = 45*.*2% (38/84); 40–49 = 22*.*6% (19/84); 50–59 = 4*.*8% (4/84); 60–80 = 1*.*2% (1/84)*	
***Gender***	*M = 69% (58/84)*, *mean age 34*.*1 y*. *F = 31% (26/84)*, *mean age 36*.*3 y*	
***ABO blood group***	*A = 33*.*3% (28/84)*, *B = 22*.*6% (19/84)*, *O = 38*.*1% (32/84)*, *AB = 6*.*0% (5/84)*	
***Diet***	*Vegetarians = 2*.*4% (2/84)*, *NO white meat = 2*.*4% (2/84)*, *NO beef = 6*.*0% (5/84)*, *NO pork = 17*.*9% (15/84)*.	
*** Vaccination history***	*Bacillus Calmette-Guerin*	*Y = 69*.*8% (58/84)*, *N = 30*.*2% (26/84)*
	*Diphtheria*, *Pertussis*, *Tetanus*	*Y = 96*.*4% (81/84)*, *N = 3*.*6% (3/84)*
	*Haemophilus influenzae type B*	*Y = 22*.*6% (19/84)*, *N = 77*.*4% (65/84)*
	*Hepatitis B*	*Y = 83*.*3% (70/84)*, *N = 16*.*7% (14/84)*
	*Influenza*	*Y = 72*.*6% (61/84)*, *N = 27*.*4% (23/84)*
	*Measles*	*Y = 89*.*3% (75/84)*, *N = 10*.*7% (9/84)*
	*Measles*, *Mumps*, *Rubella*	*Y = 90*.*5% (76/84)*, *N = 9*.*5% (8/84)*
	*Oral Poliomyelitis Vaccine*	*Y = 88*.*1% (74/84)*, *N = 11*.*9% (10/84)*
	*Typhoid*	*Y = 25*.*0% (21/84)*, *N = 75*.*0% (63/84)*
	*Varicella Zoster*	*Y = 35*.*7% (30/84)*, *N = 64*.*3% (54/84)*
	*Yellow Fever*	*Y = 20*.*2% (17/84)*, *N = 79*.*8% (67/84)*
***Geographic location during childhood***	*Europe*: *17*.*9% (15/84); Middle-East*: *13*.*1% (11/84); Africa*: *2*.*4% (2/84); South-West Asia*: *8*.*3% (7/84); East Asia*: *32*.*1% (27/84); South America*: *9*.*5% (8/84); North America*: *16*.*7% (14/84)*	

M = male; F = female; Y = yes; N = no.

Decomplementation of serum was carried out by heat inactivation for 30min at 56°C. The sera were stored at -80°C. Pooled healthy human sera (including all ABO blood groups) were used for a preliminary study.

### Sources of pig red blood cells (pRBCs) and pig aortic endothelial cells (pAECs)

Blood was obtained from a GTKO pig expressing the human complement-regulatory protein, CD46 (GTKO/CD46), and from a GTKO/CD46 pig not expressing Neu5Gc (GTKO/CD46/Neu5GcKO) (both from Revivicor, Blacksburg, VA). Both pigs were of blood type non-A (O). Cells from the same two pigs were employed in all experiments.

RBCs (which express carbohydrate antigens but do not express swine leukocyte antigens [SLA]) were isolated as previously described [24]. Briefly, RBCs were separated from whole blood, washed x3 with phosphate-buffered saline (PBS, Invitrogen, Carlsbad, CA), and centrifuged at 700*g* for 5min at 4°C. The washed RBCs were suspended in staining buffer (PBS containing 1% bovine serum albumin [Invitrogen] and 0.1% NaN_3_) for IgM/IgG binding assays.

GTKO/CD46 and GTKO/CD46/Neu5GcKO pAECs (which express both carbohydrate antigens and SLA) were isolated from pig aortas as previously described [23, 25]. Briefly, AECs were obtained from freshly-harvested pig aortas by treatment with 0.05% collagenase B (Roche Applied Science, Indianapolis, IN). The cells were collected and washed with washing medium (RPMI containing 10% heat-inactivated bovine serum [Invitrogen]) to inactivate the collagenase, and then cultured in pAEC culture medium 199 (Invitrogen) containing 10% heat-inactivated fetal bovine serum (FBS, Sigma, St. Louis, MO), antibiotic-anti mycotic (Invitrogen), and endothelial growth factor (BD Biosciences, San Jose, CA).

#### Surface staining of Gal, Neu5Gc, and hCD46 on RBCs and AECs

Surface expression of Gal, Neu5Gc, and hCD46 was determined by flow cytometry, as previously described [22, 23].

#### Binding of human serum IgM and IgG to pRBCs and pAECs by flow cytometry

Binding of human antibodies to pig cells was measured by flow cytometry using the relative geometric mean (rGM), which was calculated by dividing the geometric mean value for each sample by the negative control, as previously described [22, 23]. Briefly, pRBCs (1x10^6^/tube) and pAECs (1x10^5^/tube) were incubated with 20μL pooled human serum for 2h at 4°C. Optimal concentrations of serum were determined by a preliminary study [26]. As negative controls, human RBCs (blood group O) and human AECs were used as target cells. Sensitized baboon serum (from a baboon that had received a GTKO pig artery patch transplant without immunosuppressive therapy [27] was used as a positive control. After incubation, cells were washed with staining buffer for removing unbinding human antibodies and were blocked with 10% goat serum (Sigma) for 20min at 4°C. After further washing with staining buffer, FITC-conjugated goat-derived anti-human IgM (mμ chain-specific) or IgG (γ chain-specific) polyclonal antibody (concentration 1:100 for pRBCs and 1:50 for pAECs; Invitrogen) was added, and the cells were incubated for 30min at 4°C. After washing with staining buffer, 200μL fixation buffer was added, and the cells allowed to sit at 4°C for 30min before adding 100μL staining buffer. Flow cytometry was carried out using LSR Fortessa (BD, San Jose, CA).

For binding to pRBCs, rGM values of >1.2 (for IgM) and >1.1 (for IgG) were considered to be positive. For binding to pAECs, rGM values of >1.3 (IgM) and >1.1 (IgG) were considered to be positive.

### ELISAs for anti-Gal and anti-Neu5Gc antibodies

Anti-Gal and anti-Neu5Gc IgM/IgG antibodies in serum were detected by ELISA, as previously described [28].

### Statistical analyses

Descriptive analysis was carried out using Kruskal-Wallis paired rank test to account for the non-normality of the data. Exploratory subgroup analysis was performed to compare the median antibody binding across different groups using Kruskal-Wallis rank tests. Spearman’s rank correlation was used to describe correlations between different combinations of antibodies and genotypes. For all analyses, groups with less than 4 observations were excluded due to low statistical power. Values are presented as mean±SEM. Differences were considered to be significant at P<0.05.

## Results

### Expression of Gal, Neu5Gc, and hCD46 on wild-type, GTKO/CD46, GTKO/CD46/Neu5GcKO pig and human RBCs and AECs

**Fig 1:**
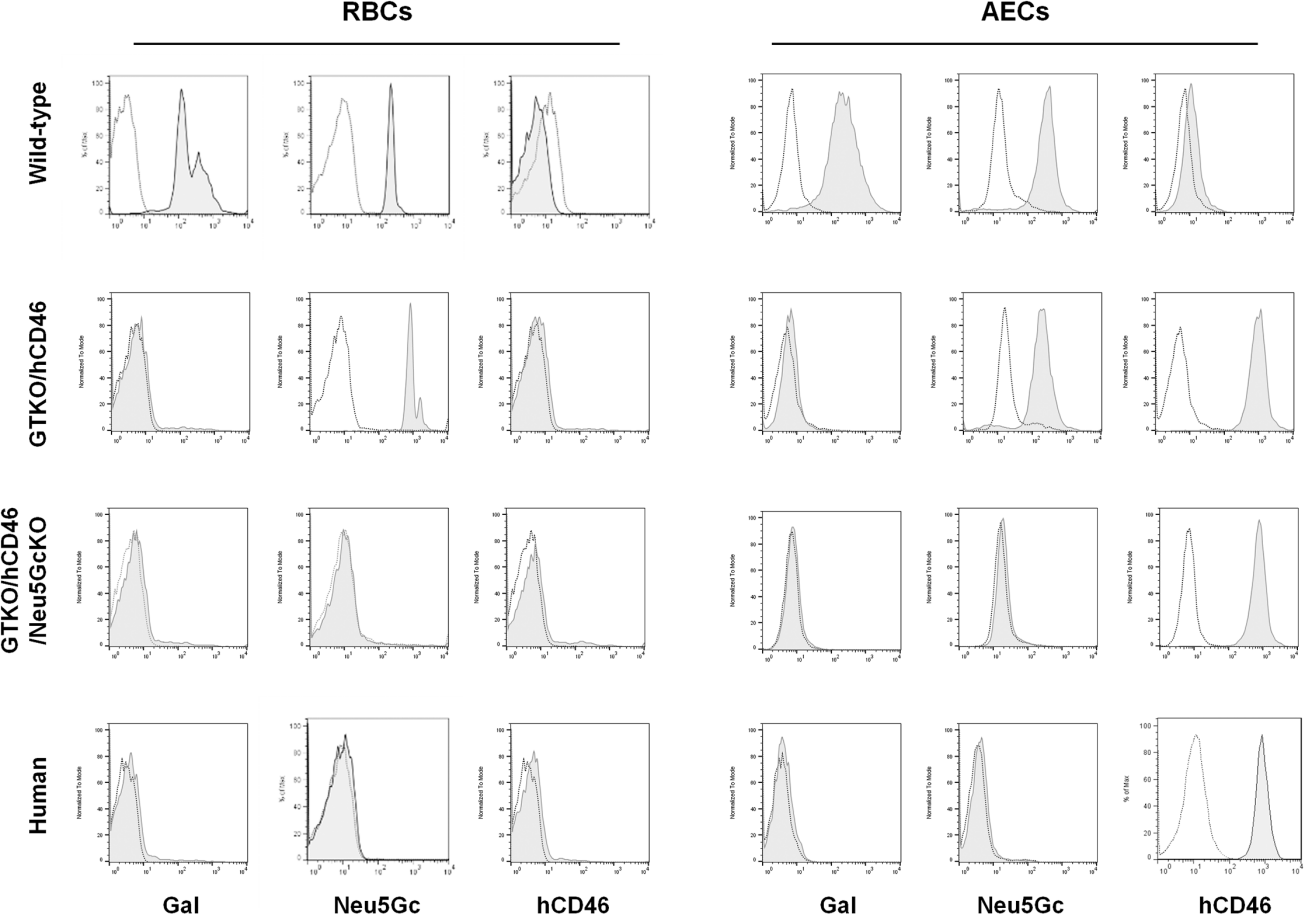
Expression of Gal, Neu5Gc, and hCD46 on wild-type, GTKO/CD46, GTKO/CD46/Neu5GcKO pig and human RBCs and AECs by flow cytometry. RBCs and AECs from GTKO/CD46 pigs expressed Neu5Gc, but not Gal. AECs from these pigs expressed hCD46, but RBCs did not. Neither Gal nor Neu5Gc was expressed on GTKO/CD46/Neu5GcKO pig RBCs or AECs. Human AECs expressed hCD46, but not Gal or Neu5Gc.

As our group has previously reported [23, 24], both Gal and Neu5Gc were expressed on wild-type pig RBCs and AECs, but hCD46 was not **(Fig 1).** RBCs and AECs from GTKO/CD46 pigs expressed Neu5Gc, but not Gal; the AECs expressed hCD46, but RBCs did not (as the transgene is not expressed in RBCs as they have no nuclei). Neither Gal nor Neu5Gc was expressed on GTKO/CD46/Neu5GcKO pig RBCs or AECs. Human AECs expressed hCD46, but not Gal or Neu5Gc. Although staining buffer which contains 1% bovine serum albumin was used for flow cytometry, it had no effect on staining for both Gal and Neu5Gc in the current and previous studies [23].

### Anti-pig IgM and IgG antibodies–definitions

**Fig 2:**
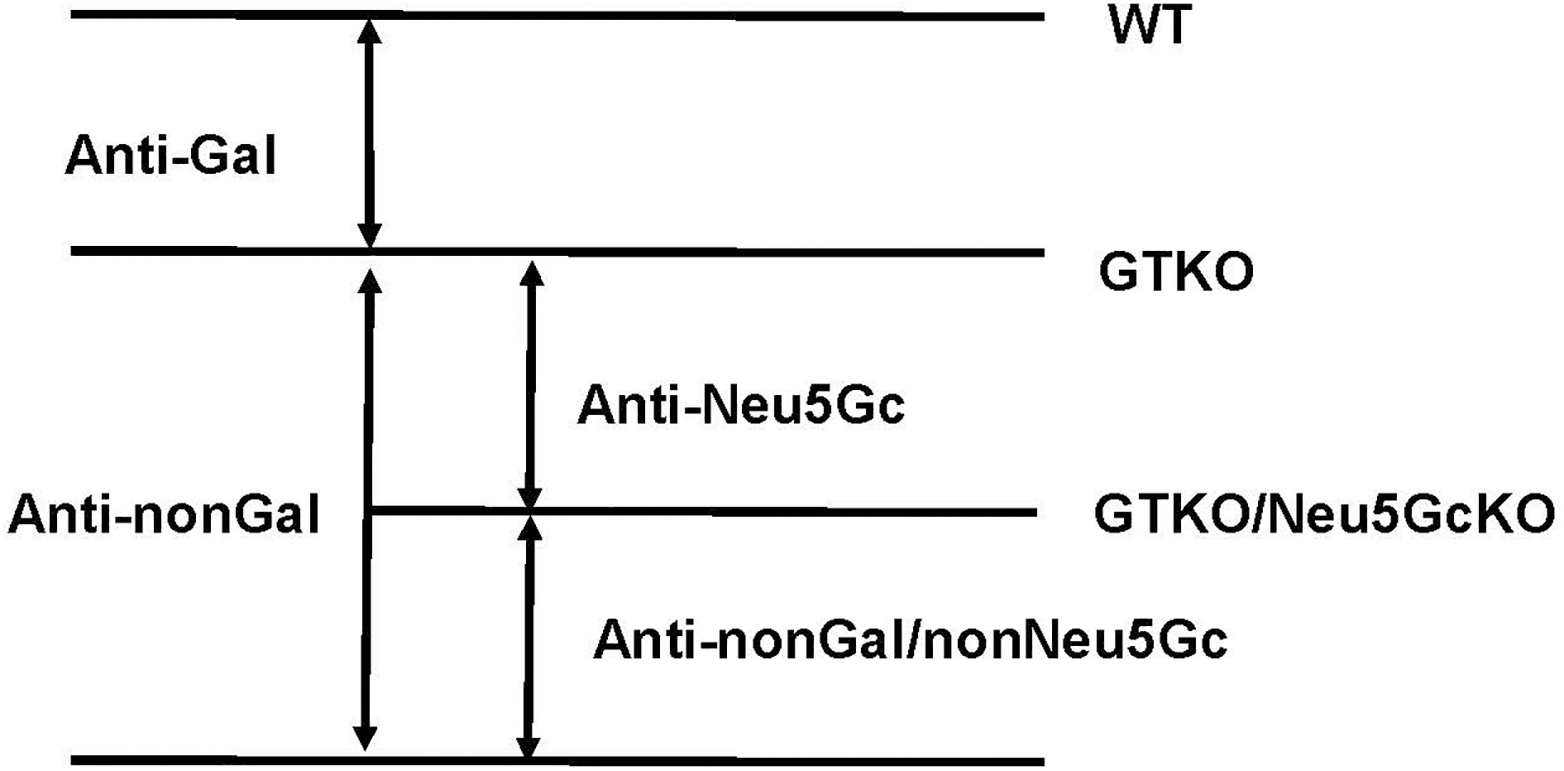
Definitions of anti-pig IgM and IgG antibodies. Anti-pig antibodies (both IgM and IgG) such as anti-Gal, anti-Neu5Gc, anti-nonGal, and anti-nonGal/nonNeu5Gc were defined according to binding to various pig cells.

For the purposes of this study, we have defined antibodies (both IgM and IgG) as follows **(Fig 2)**.

1. Anti-Gal antibody = antibody that binds to wild-type pig cells, but not to GTKO pig cells.
2. Anti-nonGal antibody = antibody that binds to GTKO pig cells.
3. Anti-Neu5Gc antibody = antibody that binds to GTKO pig cells, but not to GTKO/Neu5GcKO pig cells.
4. Anti-nonGal/nonNeu5Gc antibody = antibody that binds to GTKO/Neu5GcKO pig cells.

(N.B. Expression of hCD46 does not affect antibody binding to the cells [25]).

### Human serum anti-Gal, anti-nonGal, anti-Neu5Gc, and anti-nonGal/nonNeu5Gc IgM and IgG binding to pRBCs and pAECs

Human serum IgM and IgG binding to wild-type pig RBC has been reported previously **[23]**, and was approximately 10-fold (IgM) and 4-fold (IgG) higher than to GTKO RBCs (not shown).

**Fig 3:**
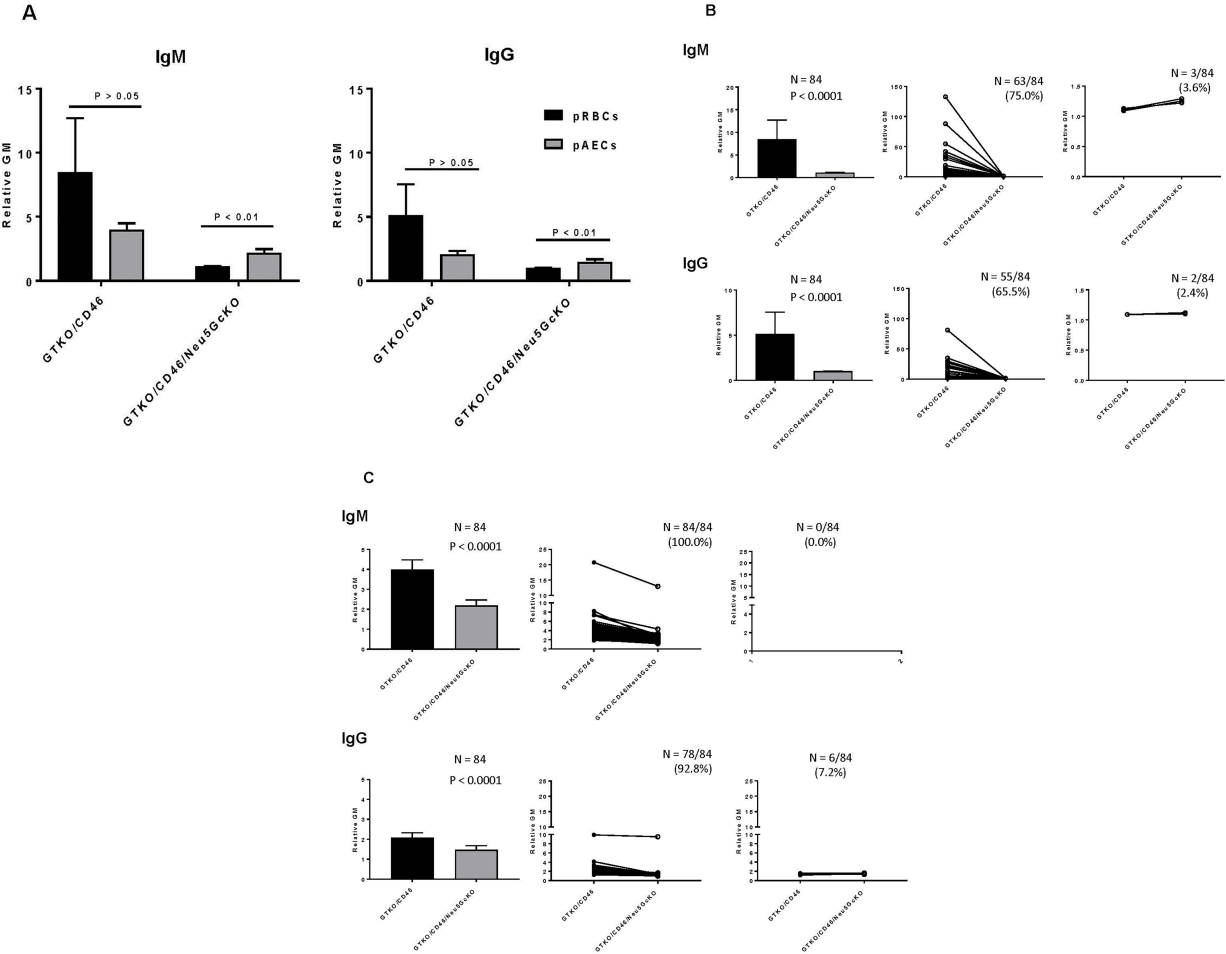
**[A] Human serum IgM/IgG binding to GTKO/CD46 and GTKO/CD46/Neu**5**GcKO pRBCs and pAECs (flow cytometry)**. There was no significant difference in IgM or IgG binding to GTKO/CD46 pRBCs or pAECs (P>0.05). IgM and IgG binding to GTKO/CD46/Neu5GcKO pRBCs were both significantly less than to the equivalent pAECs (P<0.01) although the levels of binding to pAECs were lower. **[B] Difference between IgM/IgG binding to GTKO/CD46 and GTKO/CD46/Neu**5**GcKO pRBCs.** There was a significant decrease in human IgM and IgG binding to GTKO/CD46/Neu5GcKO pRBCs when compared with binding to GTKO/CD46 pRBCs (P<0.01). IgM binding decreased in 75% of sera (63/84), though a very small number (3.6%, 3/84) were found to have very slightly increased (statistically insignificant) binding; the relative GM decreased from >8 to <1. IgG binding decreased in 65% of sera (55/84), and increased minimally in 2.4% (2/84); the relative GM was reduced from >5 to <1. **[C] Difference between IgM/IgG binding to GTKO/CD46 and GTKO/CD46/Neu**5**GcKO pAECs.** There was significantly less IgM and IgG binding to cells from GTKO/CD46/Neu5GcKO pAECs than to GTKO/CD46 pAECs (p<0.01). IgM binding was reduced in 100% of sera, and IgG in 93% of sera (78/84). Relative GM fell from 4 to 2 (IgM) and from 2 to 1.5 (IgG). No sera showed an increase in IgG binding to GTKO/CD46/Neu5GcKO cells, but 7.2% (6/84) showed no change in binding.

Human serum IgM and IgG binding to GTKO/CD46 RBCs were both *greater* than to the equivalent AECs, but the difference was not significant **(Fig 3A).** Human serum IgM and IgG binding to GTKO/CD46/Neu5GcKO RBCs were both *less* than to the equivalent AECs (P<0.01).

#### Antibody binding to pRBCs

When serum antibody binding to GTKO/CD46 pRBCs was measured, 63 of 84 sera (75%) were found to have anti-nonGal IgM, and 55 (66%) had anti-nonGal IgG **(Table 2).** This indicated that some members of the population have only anti-Gal IgM and/or anti-Gal IgG that bind to pRBCs.

**Table 2:** Incidence (and percentage) of human serum IgM and IgG antibodies binding to pRBCs and pAECs.

*Target cells*	*IgM Incidence (%)*	*IgG Incidence (%)*
***pRBCs***	*Anti-nonGal*	*Anti-nonGal*
	*63/84 (75%)*	*55/84 (66%)*
	*Anti-nonGal/nonNeuGc*	*Anti-nonGal/nonNeuGc*
	*19/84 (23%)*	*12/84 (14%)*
***pAECs***	*Anti-nonGal*	*Anti-nonGal*
	*84/84 (100%)*	*84/84 (100%)*
	*Anti-nonGal/nonNeuGc*	*Anti-nonGal/nonNeuGc*
	*82/84 (98%)*	*80/84 (95%)*

The number of sera that contained IgM that bound to GTKO/CD46/Neu5GcKO pRBCs fell to 23% (19/84), and the number that had IgG fell to 14% (12/84) **(Table 2),** indicating that a minority of the population have IgM and IgG that bind to pRBCs that express neither Gal nor Neu5Gc, i.e., are anti-nonGal/nonNeu5Gc antibodies.

#### Antibody binding to pAECs

When serum antibody binding to GTKO/CD46 pAECs was measured, all 84 sera (100%) were found to have anti-nonGal IgM and IgG **(Table 2).** This indicated that none of the population has only anti-Gal IgM or IgG that bind to pAECs.

The number of sera that contained IgM that bound to GTKO/CD46/Neu5GcKO pAECs was 82/84 (98%), and the number that contained IgG that bound to these cells was 80/84 (95%) **(Table 2).** This indicated that, in contrast to pRBCs, almost 100% of the population have IgM and IgG directed to antigens expressed on pAECs that are neither Gal nor Neu5Gc.

#### Differences between IgM/IgG binding to GTKO/CD46 and GTKO/CD46/Neu5GcKO pRBCs and pAECs

As noted above, there was a significant decrease of human IgM and IgG binding to GTKO/CD46/Neu5GcKO pRBCs when compared with binding to GTKO/CD46 pRBCs (P < 0.0001) **(Fig 3B).** IgM binding decreased significantly in 75% of sera (63/84) (P < 0.0001), though a very small number (3.6%, 3/84) were found to have non-significant increased binding. The remaining sera showed no change in IgM binding. The rGM decreased from >8 to <2. IgG binding decreased significantly in 66% of sera (55/84) (P < 0.0001), and increased non-significantly in 2.4% (2/84) **(Fig 3B)**; the rGM was reduced from >5 to <2.

When pAECs were the target cells (rather than pRBCs), IgM binding was reduced in 100% of sera (p<0.0001) **(Fig 3C),** and IgG in 93% of sera (78/84) (p<0.0001). Relative GM fell from >4 to <2 (IgM) and from >2 to <1.5 (IgG). No sera showed an increase in IgG binding to GTKO/CD46/Neu5GcKO cells, but 7% (6/84) showed no significant change in binding **(Fig 3C)**.

We conclude that the great majority of humans have anti-Neu5Gc IgM and IgG antibodies, and that there is significantly less binding to pig cells that do not express either Gal or Neu5Gc compared with those that do not express Gal alone. The level of both IgM and IgG binding to GTKO/CD46/Neu5GcKO pRBCs and pAECs was extremely low, i.e., the levels of anti-nonGal/nonNeu5Gc antibody were very low. There was more anti-Neu5Gc antibody binding to pAECs than to pRBCs, suggesting that some may be directed to non-glycan antigens.

#### Human anti-Neu5Gc IgM and IgG antibodies

**Fig 4:**
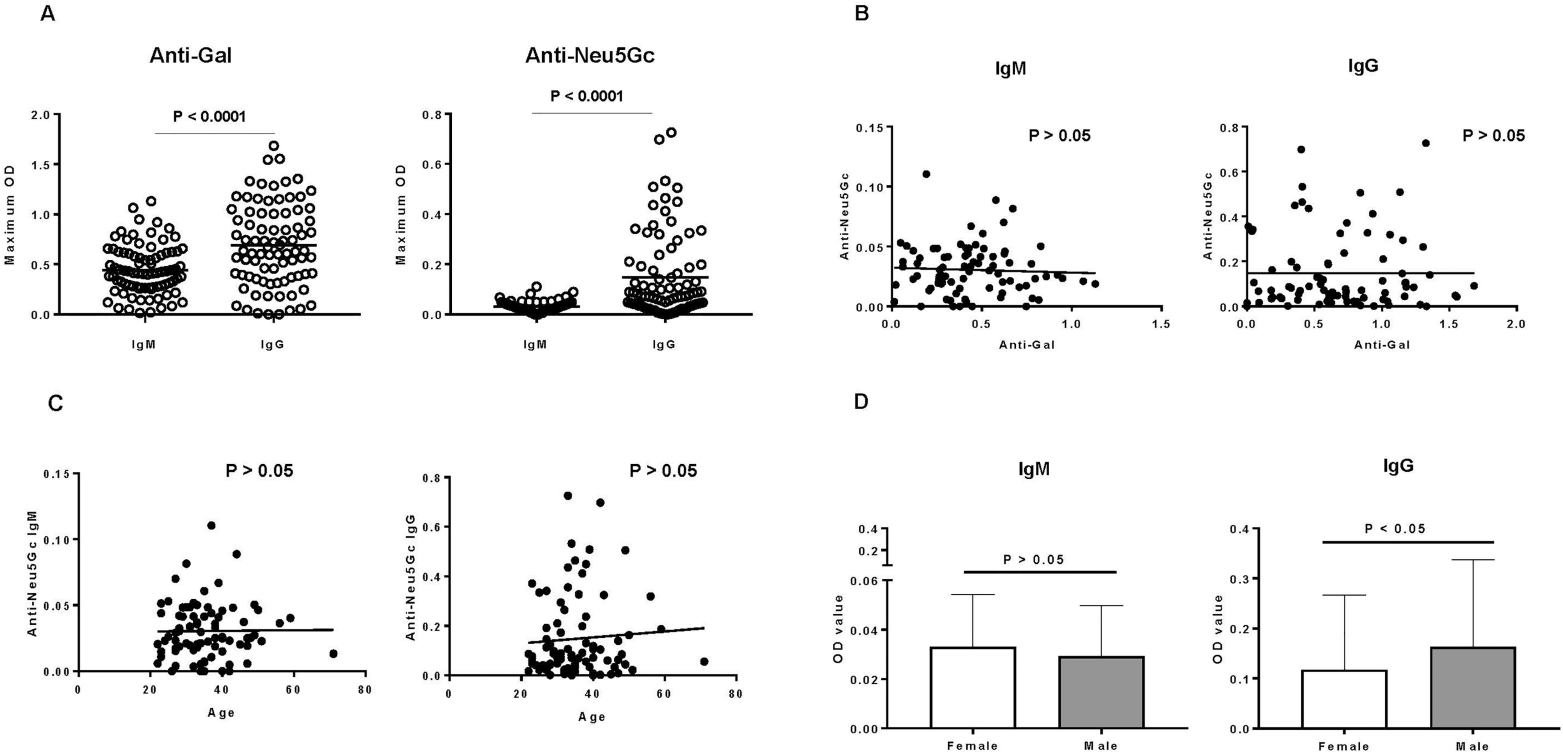
**[A] Human IgM/IgG binding to Gal and Neu**5**Gc (ELISA).** The level of anti-Gal IgM/IgG antibodies were higher than those of anti-Neu5Gc antibodies (P<0.0001). The level of anti-Gal and Neu5Gc IgG was significantly higher than that of anti-Gal and anti-Neu5Gc IgM (P<0.0001). **[B] Correlation between human serum anti-Gal and anti-Neu**5**Gc IgM and IgG antibody levels.** There is no correlation between anti-Gal level and anti-Neu5Gc level both in IgM and IgG (P>0.05). **[C] Correlation of human serum anti-Neu**5**Gc IgM and IgG antibody with age (ELISA).** There was no correlation between either anti-Neu5Gc IgM or IgG levels with age (P>0.05). **[D] Difference in human anti-Neu**5**Gc antibody between gender (ELISA).** There was no significant difference in anti-Neu5Gc IgM levels between female and male (P>0.05), but the level of anti-Neu5Gc IgG in males was significantly higher than in females (P<0.05).

Taking the study population as a whole, the level of anti-Gal and anti-Neu5Gc IgG was significantly higher than that of anti-Gal and anti-Neu5Gc IgM (P<0.0001) **(Fig 4A).** The level of anti-Gal IgM/IgG antibodies were higher than those of anti-Neu5Gc antibodies.

#### Correlation of anti-Gal with anti-Neu5Gc antibodies (ELISA)

There was no correlation between the levels of anti-Gal and anti-Neu5Gc IgM or IgG **(Fig 4B).** This suggested that the development of anti-Gal antibodies is not closely related to that of anti-Neu5Gc antibodies (even though both are ‘natural’ antibodies, and presumably have a similar mechanism of development, e.g., by exposure to gastrointestinal flora).

#### Correlation of human anti-Neu5Gc antibody with age or gender (ELISA)

There was no correlation between either anti-Neu5Gc IgM or IgG with age **(Fig 4C)**.

There was no significant difference in anti-Neu5Gc IgM levels between females and males, but the mean level of anti-Neu5Gc IgG in females was significantly lower than that of males (P<0.05) **(Fig 4D)**.

#### Correlation of human anti-Neu5Gc antibody with ABO blood group (ELISA)

There was no significant difference in anti-Gal or anti-Neu5Gc IgM/IgG level among different ABO blood groups **(S1A Fig)**.

#### IgM/IgG binding of sera of various ABO blood groups to GTKO/CD46 and GTKO/CD46/Neu5GcKO pig cells (FCM)

When GTKO/CD46 pRBCs were the target cells, the level of anti-nonGal IgG in the serum of subjects of blood group B was significantly higher than that in subjects of blood groups A, AB, and O (P<0.01) **(S1B Fig).** The levels of binding of anti-nonGal IgM and anti-nonGal/nonNeu5Gc IgM and IgG in sera of different ABO blood groups were not significantly different.

When using pAECs as target cells, there was no significant difference in binding of either anti-nonGal or anti-nonGal/nonNeu5Gc IgM/IgG to pAECs, irrespective of the ABO blood group of the serum **(S1C Fig)**.

#### Correlation of human anti-Neu5Gc antibody with diet (ELISA)

The numbers of subjects who were vegetarians (n = 2) or did not consume white meat (n = 2) were too small to be considered in a statistical analysis **(S2 Table).** There were no significant differences in the levels of anti-Neu5Gc IgM or IgG between subjects who ate a full diet and those that did not eat beef or pork.

#### Correlation of human anti-Neu5Gc antibody with previous vaccination (ELISA)

The number of subjects who had received diphtheria/pertussis/tetanus (n = 3) was too small for statistical analysis **(Table 3).** Subjects who had received Bacillus Calmette Guerin or hepatitis B vaccines had significantly *higher* serum anti-Neu5Gc IgG than those who had not received these vaccines (P<0.05). Subjects who had received a typhoid vaccine (75%) had *lower* anti-Neu5Gc IgG levels than those who had (P<0.01).

**Table 3:** Correlation of human serum anti-Neu5Gc IgM and IgG antibodies with vaccination history.

*Vaccinations received*						
*Vaccine*	*Anti-NeuGc IgM (N)*	*Anti-NeuGc IgM (Y)*	*P Value*	*Anti-NeuGc IgG (N)*	*Anti-NeuGc IgG (Y)*	*P Value*
***Bacillus Calmette Guerin***	*0*.*03 ± 0*.*02*	*0*.*03 ± 0*.*02*	*0*.*25*	*0*.*09 ± 0*.*11*	*0*.*17 ± 0*.*18*	***P<0*.*05***
*Diphtheria, *Pertussis*, *Tetanus*	*0*.*04 ± 0*.*01*	*0*.*03 ± 0*.*02*	*0*.*42*	*0*.*12 ± 0*.*06*	*0*.*15 ± 0*.*17*	*0*.*49*
***Haemophilus influenzae type B***	*0*.*03 ± 0*.*02*	*0*.*03 ± 0*.*02*	*0*.*88*	*0*.*15 ± 0*.*17*	*0*.*13 ± 0*.*15*	*0*.*41*
***Hepatitis B***	*0*.*03 ± 0*.*02*	*0*.*03 ± 0*.*02*	*0*.*84*	*0*.*08 ± 0*.*13*	*0*.*16 ± 0*.*17*	***P<0*.*05***
***Infuenza***	*0*.*03 ± 0*.*01*	*0*.*03 ± 0*.*02*	*0*.*87*	*0*.*15 ± 0*.*18*	*0*.*15± 0*.*16*	*0*.*94*
***Measles***	*0*.*03 ± 0*.*01*	*0*.*03 ± 0*.*02*	*0*.*47*	*0*.*17 ± 0*.*14*	*0*.*15 ± 0*.*17*	*0*.*3*
***Measles*, *Mumps*, *Rubella***	*0*.*03 ± 0*.*01*	*0*.*03 ± 0*.*02*	*0*.*5*	*0*.*16 ± 0*.*12*	*0*.*15 ± 0*.*17*	*0*.*34*
***Oral Poliomyelitis***	*0*.*03 ± 0*.*01*	*0*.*03 ± 0*.*02*	*0*.*74*	*0*.*15 ± 0*.*14*	*0*.*15± 0*.*17*	*0*.*88*
***Typhoid***	*0*.*03 ± 0*.*02*	*0*.*03 ± 0*.*02*	*0*.*4*	*0*.*18 ± 0*.*18*	*0*.*04 ± 0*.*05*	***P<0*.*01***
***Varicella Zoster***	*0*.*03 ± 0*.*02*	*0*.*03 ± 0*.*02*	*0*.*64*	*0*.*16 ± 0*.*17*	*0*.*13 ± 0*.*15*	*0*.*72*
***Yellow Fever***	*0*.*03 ± 0*.*02*	*0*.*03 ± 0*.*01*	*0*.*37*	*0*.*15 ± 0*.*17*	*0*.*12 ± 0*.*17*	*0*.*13*

N = No; Y = Yes

Anti-Neu5Gc IgM/IgG ELISA data are shown in terms of mean OD+/-SD (p value), which was calculated using mean OD minus negative control OD.

Bacillus Calmette Guerin and Heptatitis B vaccines: anti-Neu5Gc IgG (N) vs anti-Neu5Gc IgG (Y) P<0.05.

Typhoid vaccine: anti-Neu5Gc IgG (N) vs anti-Neu5Gc IgG (Y) P<0.01.

*The number who had received the diphtheria/pertussis/tetanus vaccine (n = 3) was too small for statistical analysis.

#### Correlation of human anti-Neu5Gc antibody levels with geographic location during childhood (ELISA)

The number of subjects brought up in Africa (n = 2) was too small for statistical analysis. There was no significant difference in anti-Neu5Gc IgM levels among subjects from different geographic locations, but subjects brought up in the Middle-East had higher levels of anti-Neu5Gc IgG than those from South West Asia, Europe, and South America (P<0.05) **(S2A Fig)**.

#### Correlation of human anti-pig IgM/IgG antibody levels with geographic location during childhood (FCM)

When the target cells were pRBCs, subjects brought up in East Asia had significantly higher anti-nonGal IgG binding than those from North America, Europe, or South America (P<0.05) **(S2B Fig).** When pAECs were the target cells, there was no significant difference among subjects from different geographic regions regarding anti-nonGal IgM and IgG levels.

There were no significant differences among subjects from different geographic locations regarding anti-nonGal/nonNeu5Gc antibody binding to pRBCs or pAECs **(S2C Fig)**.

### Correlation between various anti-pig antibody levels

#### Anti-nonGal and anti-nonGal/nonNeu5Gc antibodies (FCM)

**Fig 5:**
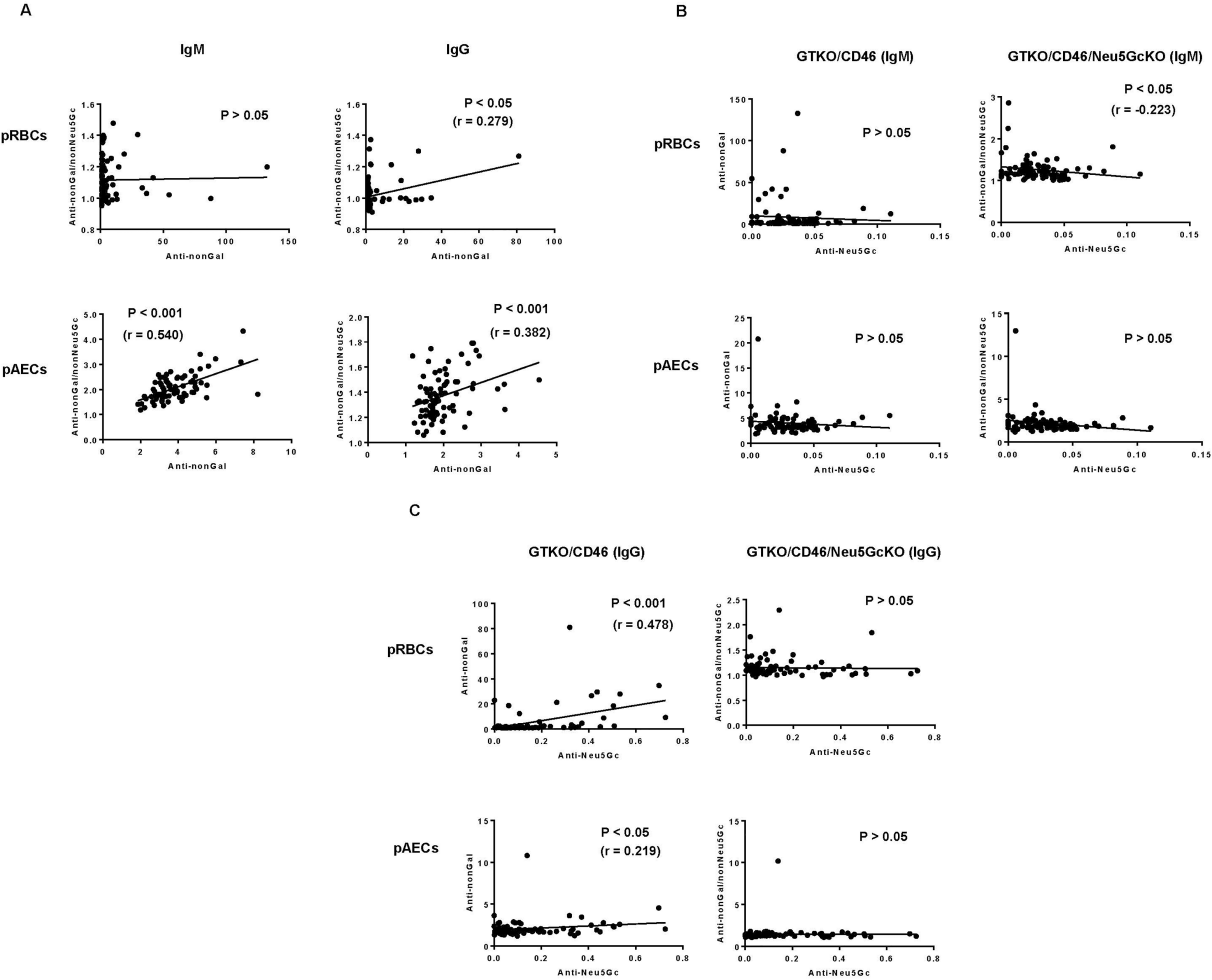
**[A] Correlation between anti-nonGal and anti-nonGal/nonNeu**5**Gc IgM and IgG antibody levels.** When pRBCs were the target cells, there was no correlation between anti-nonGal IgM and anti-nonGal/nonNeu5Gc IgM (P>0.05). There was significant positive correlation between anti-nonGal IgG and anti-nonGal/nonNeu5Gc IgG (P<0.05). When pAECs were the target cells, there was highly significant positive correlation between anti-nonGal and anti-nonGal/nonNeu5Gc IgM/IgG (P<0.001). **[B] Correlation between anti-Neu**5**Gc, anti-nonGal, and anti-nonGal/nonNeu**5**Gc IgM.** When pRBCs were the target cells, there was a significant negative correlation between anti-Neu5Gc IgM and anti-nonGal/nonNeu5Gc IgM (P<0.05), but no correlation between anti-Neu5Gc IgM and anti-nonGal IgM. When pAECs were the target cells, there was no correlation between anti-nonGal IgM and anti-nonGal/nonNeu5Gc IgM (P>0.05). **[C] Correlation between anti-Neu**5**Gc, anti-nonGal, and anti-nonGal/nonNeu**5**Gc IgG.** When pRBCs were the target cells, there was a highly significant positive correlation between anti-Neu5Gc IgG and anti-nonGal IgG (P<0.001). When pAECs were the target cells, there was a significant positive correlation between anti-Neu5Gc IgG and anti-nonGal IgG (P<0.05). There is no correlation between anti-Neu5Gc IgG and anti-nonGal/nonNeu5Gc IgG both to pRBCs and pAECs (P>0.05).

There was a significant *positive* correlation between binding of anti-nonGal IgG and anti-nonGal/nonNeu5Gc IgG to pRBCs (P<0.05), but not of IgM **(Fig 5A)**. When pAECs were the target cells, there was a significant *positive* correlation between binding of anti-nonGal and anti-nonGal/nonNeu5Gc IgM/IgG (P<0.001). These results suggested that subjects who have high levels of antibody binding to GTKO cells, might also be expected to demonstrate high levels of anti-nonGal/nonNeu5Gc antibodies.

#### Anti-Neu5Gc and anti-nonGal or anti-nonGal/nonNeu5Gc antibodies (FCM)

When pRBCs were the target cells, there was a significant *negative* correlation between anti-Neu5Gc IgM and anti-nonGal/nonNeu5Gc IgM binding (P<0.05), i.e., subjects with *low* levels of anti-Neu5Gc IgM had *high* levels of anti-nonGa/nonNeu5Gc IgM **(Fig 5B)**, suggesting that there was no correlation between anti-Neu5Gc IgM and anti-nonGal IgM binding. When pAECs were the target cells, there was no correlation between anti-nonGal IgM and anti-nonGal/nonNeu5Gc IgM binding.

When pRBCs were the target cells, there was a highly significant *positive* correlation between anti-Neu5Gc IgG and anti-nonGal IgG (P<0.001) **(Fig 5C).** When pAECs were the target cells, there was a significant *positive* correlation between anti-Neu5Gc IgG and anti-nonGal IgG (P<0.05). These results indicated that subjects who have high levels of anti-Neu5Gc antibodies also have high levels of binding to GTKO pig cells (which, of course, express Neu5Gc).

In contrast, there was no correlation between anti-Neu5Gc IgG and anti-nonGal/nonNeu5Gc IgG to either pRBCs or pAECs, suggesting that the development of anti-Neu5Gc antibodies is not associated with that of anti-nonGal/nonNeu5Gc antibodies.

## Discussion

Natural antibodies, such as anti-Gal antibodies, are detectable in the serum of healthy humans and other mammalian species before deliberate immunization. They are largely associated with exposure to glycans expressed on flora in the gastrointestinal tract [29]. In humans, the generation of anti-Neu5Gc antibodies occurs during the first year of life (by 6 months), and reaches almost adult levels at 12 months [30]. Both infant IgM and IgG anti-Neu5Gc antibodies increase soon after the introduction of Neu5Gc in the diet in the form of cow’s milk formula and baby foods containing red meat such as beef, pork, and lamb which contain high amounts of Neu5Gc. Dietary Neu5Gc is incorporated by a common human commensal bacterium, non-typeable Haemophilus influenzae, resulting in expression of Neu5Gc as an immunogenic antigen in humans, and this stimulates production of anti-Neu5Gc antibodies [30]. Natural antibodies contrast with elicited antibodies that are produced in response to direct exposure to graft antigens.

Study of a Taiwanese population, Chao et al. [31] also found no correlation between diet and anti-pig antibody levels. In addition, a study of a Korean population demonstrated that IgM andIgG antibodies against Neu5Gc were not significantly different among subjects of various blood groups and age [32].

To summarize the major observations of the present study, (i) the great majority (almost 100%) of humans had anti-Neu5Gc IgM and IgG antibodies that bind to pAECs and approximately 50% had anti-Neu5Gc antibodies that bind to pRBCs, (ii) there was significantly less human antibody binding to pig cells that do not express either Gal or Neu5Gc compared with those that do not express Gal alone, (iii) subjects of blood group B had higher levels of IgG binding to nonGal pRBCs than those of other blood groups (cause uncertain), (iv) the levels of both IgM and IgG binding to GTKO/CD46/Neu5GcKO pRBCs and pAECs were low, (v) the level of anti-Neu5Gc IgG was higher in men than women, (vi) the level did not change with age or diet, and there was some variability associated with (vii) previous vaccination history and (viii) the geographic region in which the individual spent his or her childhood. Increasing the sample size would be necessary to draw significant conclusions.

(As all subjects had been living in the USA for periods of time ranging from a minimum of 6 months to a maximum of 48 years, their gastrointestinal flora may have changed [which may have influenced the level of anti-Neu5Gc antibodies], although the microorganisms to which a subject is exposed during childhood, including gastrointestinal tract flora, may possibly influence antibody production throughout life).

In the present study, we investigated the correlation of human serum anti-Neu5Gc antibodies with each individual vaccination history, rather than combination of vaccinations **(Table 3, S1 Table)** because of the small numbers. Therefore, it is uncertain whether the level of anti-Neu5Gc antibodies might also be associated with the combination of vaccines. In addition, it is unknown whether these vaccinations include Neu5Gc antigen. Although some minor differences in the level of anti-Neu5Gc antibody were found between those exposed to vaccines (e.g., typhoid) and those not, the relevance of these observations is uncertain.

It has been shown that the post-natal appearance of anti-carbohydrate antibodies, such as anti-Gal [29], anti-Neu5Gc [30], and anti-blood group [33] antibodies, is elicited by colonizing gut bacteria expressing these epitopes during infancy in humans. Therefore, we anticipated that there would be a positive correlation between anti-Gal and anti-Neu5Gc antibody levels in humans. However, the present study demonstrated that there was no significant correlation between the levels of these antibodies. There were positive correlations (i) between anti-nonGal and anti-nonGal/nonNeu5Gc antibody levels, and (ii) between anti-nonGal and anti-Neu5Gc levels, but not between anti-Neu5Gc and anti-nonGal/nonNeu5Gc antibody levels. It will be important to understand the mechanism of the development of anti-nonGal/nonNeu5Gc antibodies as well as the time of their emergence in humans, although the levels of anti-nonGal/nonNeu5Gc antibodies are much lower than anti-nonGal antibodies.

One of the limitations of the current study was our method for detecting anti-Neu5Gc antibody by ELISA. Anti-Neu5Gc antibodies bind to several forms of Neu5Gc [34]. The Neu5Gc-PAA conjugate which was used for ELISA in the present study does not present a sufficiently diverse range of antigens to accurately measure the amount of anti-Neu5Gc in the serum, and therefore does not give a complete comparison of the antibody levels in humans. In future experiments, it will be necessary to use more pertinent methodologies for measuring anti-Neu5Gc antibodies [30, 32, 35, 36].

As pigs that do not express Gal or Neu5Gc (or S[d]a [37]) are most likely to be the sources of organs and cells for clinical xenotransplantation, perhaps the most important observation in the present study is the low levels of IgM and IgG in the sera directed to nonGal/nonNeu5Gc antigens. As serum binding to pRBCs reflects only the importance of antibodies to glycan epitopes, our data on binding to pAECs (that may also reflect binding to SLA) is probably more relevant to the clinical development of pig organ xenotransplantation. Although the binding to pAECs was very low, almost 100% of the humans tested had serum IgM and IgG directed to antigens expressed on pAECs that were neither Gal nor Neu5Gc. Many of these antibodies were likely directed to S(d)a [21, 37], but a minority may have been directed to unknown pig antigens [38].

Our observation of the positive correlation between binding (to pAECs) of anti-nonGal IgM and IgG and of anti-nonGal/nonNeu5Gc IgM and IgG suggests that S(d)a may be a significant target for human anti-pig antibodies. Whether, in the presence of human complement- and/or coagulation-regulatory proteins expressed on the pig vascular endothelium, such a low level of binding is clinically important remains uncertain, but knockout of the gene for β1,4 N-acetylgalactosaminyltransferase (that is responsible for S[d]a expression) will almost certainly be an advantage [21].

Although there were some limitations to this study, e.g., the number of samples was relatively small, we believe that the data provide valuable information on the incidence and levels of antibodies to Neu5Gc and to nonGal/nonNeu5Gc pig antigens. The data may also prove valuable in efforts to modify pigs so that pRBCs could be used for clinical transfusion in the future [39–41].

## Supporting information

S1 Fig **[A] Binding of human serum anti-Gal and anti-Neu5Gc IgM and IgG antibodies of various ABO blood groups.**
There was no significant differences in anti-Gal or anti- Neu5Gc IgM/IgG levels with ABO blood group (P>0.05).
**[B] IgM/IgG binding of human sera of various ABO blood groups to GTKO/CD46 and GTKO/CD46/Neu5GcKO pRBCs**.
The level of anti-nonGal IgG of blood group B was significantly higher than that of blood group O, AB, and A (P<0.01). There were no differences in anti-nonGal IgM levels among blood groups O, AB, and A. The levels of anti-nonGal IgM and anti-nonGal/nonNeu5Gc IgM among different blood groups were not significantly different.
**[C] IgM/IgG binding of sera of various ABO blood groups to GTKO/CD46 and GTKO/CD46/Neu5GcKO pAECs.**
There was no significant difference in either anti-nonGal or anti-nonGal/nonNeu5Gc IgM/IgG levels in subjects with different blood groups (P>0.05).
(TIF)

S2 Fig **[A] Difference of human anti-Gal and anti-Neu5Gc antibody with geographic location.**
The numbers of Africa are too small to consider in statistical analysis (n = 2).
There is no significant difference in anti-Gal IgM and anti-Neu5Gc IgM as well as anti-Gal IgG levels among different geographical locations (P>0.05). There is a significant difference in anti-Neu5Gc IgG levels among different geographical areas. Subjects of Middle-East had higher levels of anti-Neu5Gc IgG than that of South West Asia, Europe and South America (P<0.05).
**[B] Difference of human anti-nonGal antibody with geographic location (pRBCs and pAECs).**
The numbers of Africa are too small to consider in statistical analysis (n = 2).
There is no significant difference among different geographic regions in anti-nonGal IgM binding to pRBCs (P>0.05). However, there is a significant difference of anti-nonGal IgG in various locations binding to pRBCs. Subjects of East Asia had significant higher anti-nonGal IgG level than that of North America, Europe and South America when binding to pRBCs (P<0.05). When using pAECs as target cells, there is no significant difference among different geographic regions regarding to anti-nonGal IgM and IgG levels (P>0.05).
**[C] Difference of human anti-nonGal/nonNeu5Gc antibody with geographic location (pRBCs and pAECs).** The numbers of Africa are too small to consider in statistical analysis (n = 2). There is no significant difference among different geographic locations regarding anti-nonGal/nonNeu5Gc antibody binding to pRBCs and pAECs (P>0.05).
(TIF)

S1 Table Statistically significant observations made from a previous study of sera from 75 healthy human subjects. (DOCX)

S2 Table Correlation of human serum anti-Neu5Gc IgM and IgG antibodies with diet. (DOCX)

## Abbreviations

AECs: aortal endothelial cells
CMAH: cytidine monophospho-N-acetylneuraminic acid hydroxylase
ELISA: enzyme-linked immunosorbent assay
FCM: flow cytometry
Gal: galactose-α1,3-galactose
GTKO: α1,3-galactosyltransferase gene-knockout
Neu5Gc: N-glycolylneuraminic acid
Neu5GcKO: monophospho-N-acetylneuraminic acid hydroxylase gene-knockout
RBCs: red blood cells
rGM: relative geometric mean
