# Supplementary figures and images for: Anti-Neu5Gc and anti-non-Neu5Gc antibodies in healthy humans
Source: PLoS One. 2017 Jul 17;12(7):e0180768. doi: 10.1371/journal.pone.0180768 (PMC5513429; doi:10.1371/journal.pone.0180768)

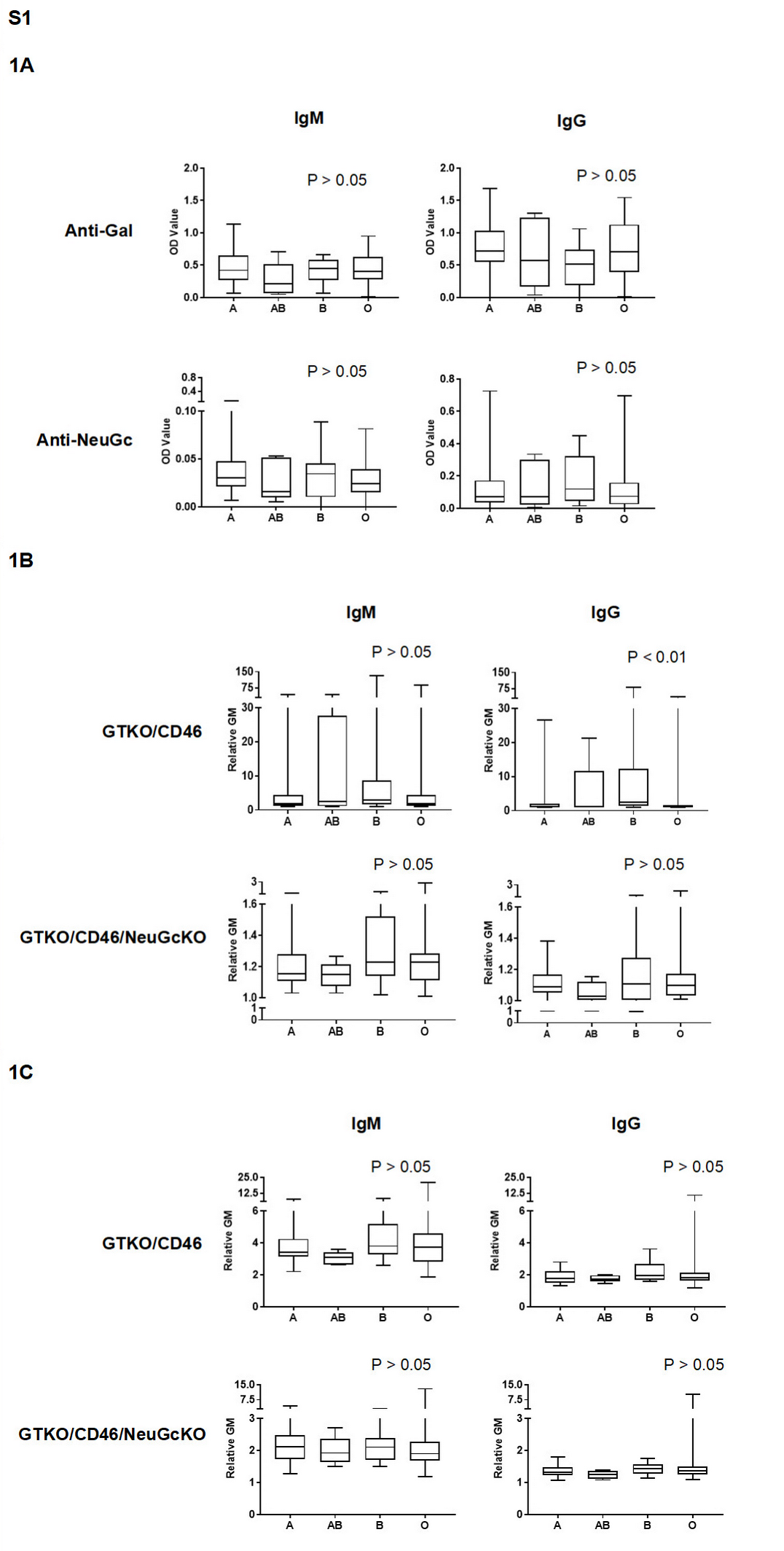

Supplement: S1 Fig — [A] Binding of human serum anti-Gal and anti-Neu5Gc IgM and IgG antibodies of various ABO blood groups. There was no significant differences in anti-Gal or anti- Neu5Gc IgM/IgG levels with ABO blood group (P>0.05). [B] IgM/IgG binding of human sera of various ABO blood groups to GTKO/CD46 and GTKO/CD46/Neu5GcKO pRBCs. The level of anti-nonGal IgG of blood group B was significantly higher than that of blood group O, AB, and A (P<0.01). There were no differences in anti-nonGal IgM levels among blood groups O, AB, and A. The levels of anti-nonGal IgM and anti-nonGal/nonNeu5Gc IgM among different blood groups were not significantly different. [C] IgM/IgG binding of sera of various ABO blood groups to GTKO/CD46 and GTKO/CD46/Neu5GcKO pAECs. There was no significant difference in either anti-nonGal or anti-nonGal/nonNeu5Gc IgM/IgG levels in subjects with different blood groups (P>0.05). (TIF) [file pone.0180768.s001.tif]

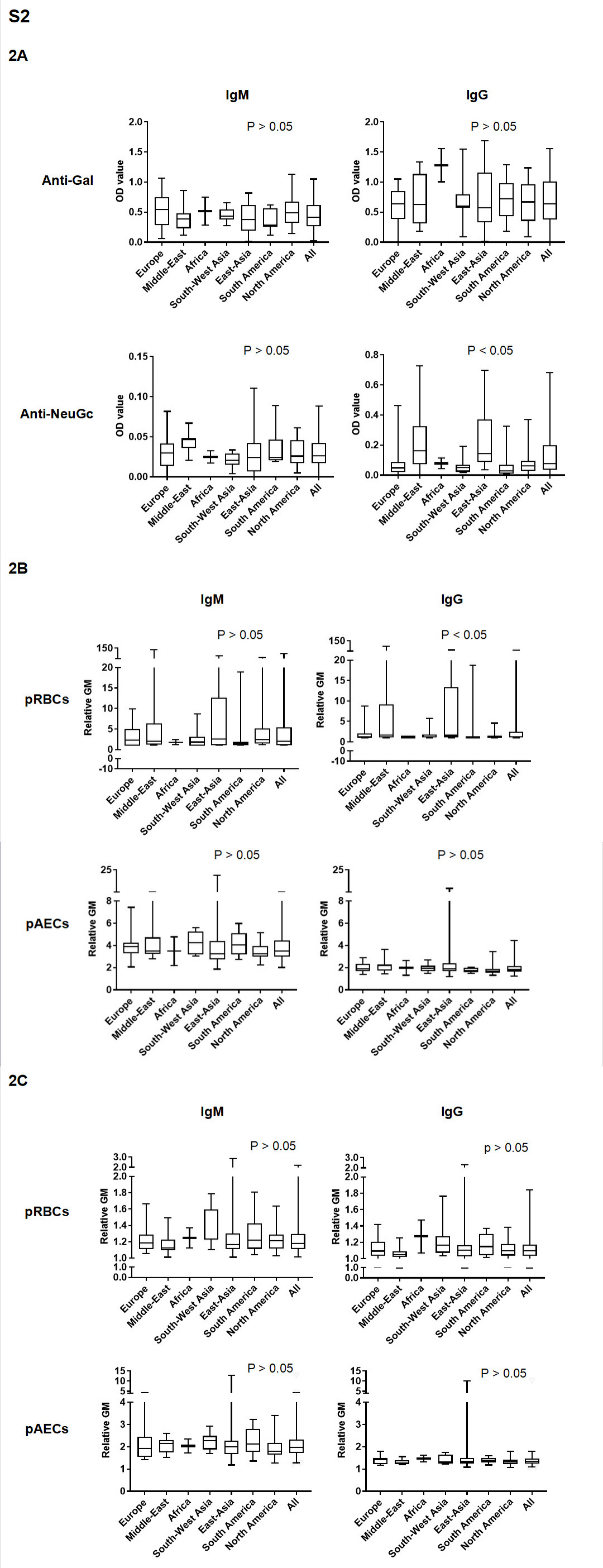

Supplement: S2 Fig — [A] Difference of human anti-Gal and anti-Neu5Gc antibody with geographic location. The numbers of Africa are too small to consider in statistical analysis (n = 2). There is no significant difference in anti-Gal IgM and anti-Neu5Gc IgM as well as anti-Gal IgG levels among different geographical locations (P>0.05). There is a significant difference in anti-Neu5Gc IgG levels among different geographical areas. Subjects of Middle-East had higher levels of anti-Neu5Gc IgG than that of South West Asia, Europe and South America (P<0.05). [B] Difference of human anti-nonGal antibody with geographic location (pRBCs and pAECs). The numbers of Africa are too small to consider in statistical analysis (n = 2). There is no significant difference among different geographic regions in anti-nonGal IgM binding to pRBCs (P>0.05). However, there is a significant difference of anti-nonGal IgG in various locations binding to pRBCs. Subjects of East Asia had significant higher anti-nonGal IgG level than that of North America, Europe and South America when binding to pRBCs (P<0.05). When using pAECs as target cells, there is no significant difference among different geographic regions regarding to anti-nonGal IgM and IgG levels (P>0.05). [C] Difference of human anti-nonGal/nonNeu5Gc antibody with geographic location (pRBCs and pAECs). The numbers of Africa are too small to consider in statistical analysis (n = 2). There is no significant difference among different geographic locations regarding anti-nonGal/nonNeu5Gc antibody binding to pRBCs and pAECs (P>0.05). (TIF) [file pone.0180768.s002.tif]
