# Supplementary material for: Anti-Neu5Gc and anti-non-Neu5Gc antibodies in healthy humans
Source: PLoS One. 2017 Jul 17;12(7):e0180768. doi: 10.1371/journal.pone.0180768 (PMC5513429; doi:10.1371/journal.pone.0180768)
Supplement: S1 Table — (DOCX) [file pone.0180768.s003.docx]

**S1 Table:** **Statistically significant observations made from a previous study of sera from 75 healthy human subjects***

| ***Antibodies*** | ***Observations*** |
| --- | --- |
| ***Anti-pig IgM*** | *Decreased with age (P<0.001), Subjects of blood group A had higher levels than those of blood group AB (P<0.005)*  *Japanese had higher levels than Europeans (P<0.05)* |
| ***Anti-pig IgG*** | *Subjects of blood group A had higher levels than those of blood group AB (P<0.05) Subjects from the Middle-East had higher levels than others, except for those from South East Asia (P<0.05)* |
| ***Anti-Gal IgM*** | *Decreased with age (P<0.001) Women had higher levels than men (P<0.05)* |
| ***Anti-Gal IgG*** | *Measles-Mumps-Rubella vaccination was associated with lower levels (P<0.05)* |
| ***Anti-nonGal IgM*** |  |
| ***Anti-nonGal IgG*** | *Increased with age (P<0.05), Vaccination against typhoid was associated with lower levels (P<0.05), Vaccination against influenza was associated with higher levels (P<0.05), Subjects of Middle-Eastern origin had higher levels than those from Europe (P<0.05)* |

*Source - reference 11
