# Supplementary material for: Anti-Neu5Gc and anti-non-Neu5Gc antibodies in healthy humans
Source: PLoS One. 2017 Jul 17;12(7):e0180768. doi: 10.1371/journal.pone.0180768 (PMC5513429; doi:10.1371/journal.pone.0180768)
Supplement: S2 Table — (DOCX) [file pone.0180768.s004.docx]

**S2 Table: Correlation of human serum anti-NeuGc IgM and IgG antibodies with diet**

| ***Diet*** | ***Anti-NeuGc***  ***IgM (N)*** | ***Anti-NeuGc***  ***IgM (Y)*** | ***P Value*** | ***Anti-NeuGc***  ***IgG (N)*** | ***Anti-NeuGc***  ***IgG (Y)*** | ***P Value*** |
| --- | --- | --- | --- | --- | --- | --- |
| ****Vegetarian*** | *0.03±0.02* | *0.02±0.01* | *0.63* | *0.15 ±0.17* | *0.04±0.03* | *0.3* |
| ***No beef*** | *0.02±0.01* | *0.03±0.02* | *0.23* | *0.17±0.2* | *0.15±0.17* | *0.97* |
| ***No pork*** | *0.03±0.02* | *0.03±0.02* | *0.79* | *0.15±0.21* | *0.15±0.16* | *0.59* |
| ****No white meat*** | *0.02±0.01* | *0.03±0.02* | *0.63* | *0.04±0.03* | *0.15±0.17* | *0.3* |

N=No; Y=Yes

Anti-NeuGc IgM/IgG ELISA data are shown as mean OD+/-SD (p value), which was calculated using mean OD minus negative control OD.

*The numbers of subjects who were vegetarians or did not consume white meat (n=2 in each group) were too small for statistical analysis.
